# Texture Descriptors Ensembles Enable Image-Based Classification of Maturation of Human Stem Cell-Derived Retinal Pigmented Epithelium

**DOI:** 10.1371/journal.pone.0149399

**Published:** 2016-02-19

**Authors:** Loris Nanni, Michelangelo Paci, Florentino Luciano Caetano dos Santos, Heli Skottman, Kati Juuti-Uusitalo, Jari Hyttinen

**Affiliations:** 1 Department of Information Engineering, University of Padua, Padua, Italy; 2 Department of Electronics and Communications Engineering, Tampere University of Technology, BioMediTech, Tampere, Finland; 3 University of Tampere, BioMediTech, Tampere, Finland; North Shore Long Island Jewish Health System, UNITED STATES

## Abstract

**Aims:**

A fast, non-invasive and observer-independent method to analyze the homogeneity and maturity of human pluripotent stem cell (hPSC) derived retinal pigment epithelial (RPE) cells is warranted to assess the suitability of hPSC-RPE cells for implantation or *in vitro* use. The aim of this work was to develop and validate methods to create ensembles of state-of-the-art texture descriptors and to provide a robust classification tool to separate three different maturation stages of RPE cells by using phase contrast microscopy images. The same methods were also validated on a wide variety of biological image classification problems, such as histological or virus image classification.

**Methods:**

For image classification we used different texture descriptors, descriptor ensembles and preprocessing techniques. Also, three new methods were tested. The first approach was an ensemble of preprocessing methods, to create an additional set of images. The second was the region-based approach, where saliency detection and wavelet decomposition divide each image in two different regions, from which features were extracted through different descriptors. The third method was an ensemble of Binarized Statistical Image Features, based on different sizes and thresholds. A Support Vector Machine (*SVM*) was trained for each descriptor histogram and the set of *SVM*s combined by sum rule. The accuracy of the computer vision tool was verified in classifying the hPSC-RPE cell maturation level.

**Dataset and Results:**

The RPE dataset contains 1862 subwindows from 195 phase contrast images. The final descriptor ensemble outperformed the most recent stand-alone texture descriptors, obtaining, for the RPE dataset, an area under ROC curve (*AUC*) of 86.49% with the 10-fold cross validation and 91.98% with the leave-one-image-out protocol. The generality of the three proposed approaches was ascertained with 10 more biological image datasets, obtaining an average *AUC* greater than 97%.

**Conclusions:**

Here we showed that the developed ensembles of texture descriptors are able to classify the RPE cell maturation stage. Moreover, we proved that preprocessing and region-based decomposition improves many descriptors’ accuracy in biological dataset classification. Finally, we built the first public dataset of stem cell-derived RPE cells, which is publicly available to the scientific community for classification studies. The proposed tool is available at https://www.dei.unipd.it/node/2357 and the RPE dataset at http://www.biomeditech.fi/data/RPE_dataset/. Both are available at https://figshare.com/s/d6fb591f1beb4f8efa6f.

## Introduction

The retinal pigment epithelial (RPE) cells reside in the back of the eye between the photoreceptor cells and choroid. The RPE monolayer is vitally important for the vision as RPE cells compose a diffusion barrier to protect photoreceptor cells from humoral substances, but also maintain the viability of photoreceptor cells [1]. The RPE cell differentiation and maturation is a slow process, modulated by culturing environmental trophic factors [2,3]. The morphology changes during maturation [4]: from the elongated, so called “fusiform morphology”, of immature RPE; via “epithelioid morphology” i.e. rounder but still without pigmentation (after one to two weeks of culture); to “cobblestone morphology” (approximately after a month) when the cells have condensed and become heavily pigmented [4]. This phenomenon can be seen both in primary RPE [4] and in human pluripotent stem cells (hPSC) derived RPE cell maturation [5]. Recently, in the first human embryonic stem cells (hESC) RPE transplantations to humans, it was demonstrated that less pigmented cells integrated better than heavily pigmented cells [6]. Furthermore, new serial plating methods to expand the hPSC-RPE cell number [7,8] need a quality and purity evaluation after every plating step [8]. These applications would benefit from a non-invasive and reliable method to assess the maturity development of hPSC-RPE cells. The benefits of cell morphology analysis for both RPE tissue [9] and hPSC-RPE cell cultures [10] has already been shown. However, this has been mainly done by manual examination and therefore is affected by inter- and intra-operator variability, making it less suitable for clinical application. In particular, Jiang *et al*. [9] recently published a computer vision approach for RPE tissue explants, discriminating between age (young, <61days-old *vs* old, ≥100 or 180 days-old) and genotype (control *vs* rd10, considered to be a model for autosomal recessive retinitis pigmentosa). The analysis was based on 21 morphological features of the cells, including aspect ratio and area, by means of principal component analysis. In [11], three degrees of pigmentation were considered as a good maturation marker. A manual approach was chosen, where two observers subjectively classified the cell pigmentation levels and an objective pigmentation measurement was inferred from the Photoshop's Info Palette for a set of manually-selected points.

In this paper, we focused on the specific problem of the classification of the maturation level of hPSC-RPE cells by means of a different approach: texture analysis. Together with the increasing availability of advanced and accurate image acquisition techniques, texture analysis has become nowadays a common processing approach for medical and biological images. Its versatility makes it applicable to images acquired with diverse modalities: from medical imaging to microscopy [12–15].

In spite of the recent progresses in texture analysis, textural information in medical images is still often assessed by conventional features such as first order statistics (e.g. variance, kurtosis and skewness), second order statistics (Haralick features extracted from the grey level co-occurrence matrix [16]) or wavelet features. In [17], wavelets and a subset of the Haralick features were extracted from X-ray images to diagnose presence of osteosarcomas. First order statistics and features extracted from the grey level co-occurrence matrix and run-length matrix proved their utility in colorectal polyp identification in colonoscopy [18], classification of intracardiac masses (thrombi, malignant, and benign tumors) for cardiac tumor detection [19] and breast cancer malignancy classification in histological images [20].

More recent techniques, such as local binary pattern (*LBP*) [21] or texture descriptors derived from it (e.g. local ternary pattern (*LTP*) [22], local quinary pattern (*LQP*) [13], etc.), were applied to medical imaging for the examination of Pap test samples [12] or in the inquiry of endoscopy images of healthy and celiac disease duodenal tissue [14]. Another important research area, where texture descriptors are commonly used, is cell classification. Due to the availability of many datasets (2D HeLa dataset (HeLa) [23], chinese hamster ovary cells (CHO) [24], etc.), this field is very prolific for specific classification tasks and for the development of more and more accurate texture descriptors. In [13], the multi-threshold approach was applied to *LTP* and *LQP* and tested by classifying six different datasets of cellular and subcellular organelles. In [15], a new variant of *LBP*, the rotation invariant co-occurrence among adjacent *LBP* (*RICLBP*), obtained outstanding results in the MIVIA HEp-2 dataset. It suggests also that clinical tests, such as the antinuclear antibody test, can benefit of improved accuracy texture descriptors.

In spite of the efforts performed during the latest years to improve the discriminant power of texture descriptors, preprocessing did not receive the same attention. Recently published preprocessing approaches exploit the separation of the texture image in two different regions or maps, e.g. textural information extracted from edge information. In [25], the Difference of Gaussians (*DoG*) filter was used to compute from a given image two maps representing the “positive” and the “negative” sides of the image edges, resulting in a classification accuracy improvement. A similar approach was exploited by [26] (details in section 2.3) for the extraction, through Sobel filtering, of an edge and a non-edge region from a texture image to compute *LBP*, *LTP*, etc. on the original image masked by each map. The technique is interesting since it can be combined with many state-of-the-art texture descriptors. In order to differentiate from the canonical preprocessing, we named the descriptors combined with this approach as region-based descriptors. In addition to them, well-assessed preprocessing algorithms were tested, e.g. wavelet [27] and Gabor filters [28]. We paid particular attention to preprocessing techniques in this study, to improve the descriptors classification power.

The main aim of this paper was to develop three simple but effective methods to create ensembles of texture descriptors: the ensemble of preprocessing, the region-based approach and the ensemble of Binarized Statistical Image Features (*Bsif*). We validated them on a wide range of biological image datasets, with particular focus on the quantification of hPSC-RPE cell maturation stages, which enables a user-independent method to analyze the cell cultures before their use in implantation or as *in vitro* cell models. To find the most suitable ensemble of descriptors for the classification of the three developmental stages, we tested a combination of large sets of both preprocessing methods and texture descriptors. In the perspective of using hPSC-RPE cells for drug tests or transplantation, we used phase-contrast microscopy images, which is a noninvasive assessment method. Our work resulted in a methodological core for a software tool in order to assess quantitatively the level of development of hPSC-RPE cells. The same ensembling methods resulted effective also for other classification problems, ranging from medical diagnostic to virus images.

The pipeline of the process consisted in the following steps. First, we considered many state-of-the-art stand-alone texture descriptors in order to select the best ones. Second, they were combined together and with techniques to augment and enhance the features extracted from each image, to improve the classification performances. Finally, the best performing features sets resulting from the previous step were combined together, thus resulting in ensembles which improved significantly the performances of the pre-existing stand-alone descriptors and of the ensembles based on a single descriptor. For classification, we used Support Vector Machines (*SVM*).

In detail, we propose the following novelties. First, an ensemble of preprocessing approaches (based on wavelet decomposition, Gabor filtering, orientation image and Multi-scale approach by Gaussian filtering) was applied to create a set of images to be used together with the original one. A different descriptor was extracted from each processed image and the set of *SVM*s combined by sum rule. Second, saliency detection and wavelet decomposition were tested for the region-based descriptors. Each image was divided into two regions from which histograms were extracted by different texture descriptors. From each histogram, a specific *SVM* is trained [29]. Finally, the partial scores obtained by the different *SVM*s were combined by sum rule. Third, the original stand-alone versions of the *Bsif* [30] was improved by combining different *Bsif* sets. They were obtained by (i) varying the size of the filter and (ii) introducing a threshold while building the *Bsif* image. We constructed a new ensemble, using a set of sizes and thresholds, which greatly outperformed the stand-alone version.

## Materials and Methods

### 2.1 Proposed approaches

In this work, we developed and validated three methods for ensembling texture descriptors and techniques such as preprocessing or region-based feature extraction, to describe images with an augmented feature set and eventually improving their classification. The first method was a combination of preprocessing methods, to create additional images from the original one and then extract texture information from each of them, enhancing the feature set describing the original image. The second approach worked differently, obtaining the augmentation of the feature set by means of the region-based approach. Saliency detection and wavelet decomposition divide each image in two different regions, from which features were extracted through different descriptors. Again, the original image is described by an enhanced feature set. Different texture descriptors were tested with the first two approaches. The third method augmented the feature set describing the original image by combining feature vectors extracted by *Bsif*, based on different filter sizes and binarization thresholds. The most effective feature sets extracted according to the aforementioned approaches were ensembled together. To test and optimize our approaches for the hPSC-RPE classification problem, we built a new RPE image dataset (Section 2.2): 1862 subwindows were extracted from 195 phase contrast images of maturing hPSC-RPE cells. Finally, we analyzed how the three approaches performed independently on the analyzed datasets: in order to generalize their viability, 10 large datasets of medical and biological images were used (Section 2.3).

The remainder of this section is organized as follows: the section 2.1.1 briefly explains the basic texture descriptors used in this paper; the section 2.1.2 is dedicated to preprocessing techniques and their ensemble; the section 2.1.3 presents the region-based approach using, saliency maps and wavelet maps; the section 2.1.4 explains the ensemble of *Bsif*; and, finally, the section 2.1.5 details the new multi-quinary coding tests.

#### 2.1.1 Texture descriptors

The standards texture descriptors used in this work are summarized in Table 1, together with the chosen parameters. As for the *LBP*-based approaches, we tested both uniform and rotation invariant uniform bins (see section 3 for details).

**Table 1:** Texture descriptors and their parameter sets.

Acronym	Descriptor and parameters	Ref
*LBP-HF*	Multi-scale *LBP* histogram Fourier features with 2 (radius, neighboring points) configurations: (1,8) and (2,16).	[31]
*LPQ*	Multi-scale local phase quantization with radius 3 and 5.	[32]
*HOG*	Histogram of oriented gradients with 30 cells (5 by 6).	[33]
*LBP*	Multi-scale uniform *LBP* with 2 (radius, neighboring points) configurations: (1,8) and (2,16).	[21]
*LTP*	Multi-scale uniform *LTP* with 2 (radius, neighboring points) configurations: (1,8) and (2,16).	[34]
*MLQP*	The multi-quinary coding version of *LBP* and its parameters as proposed in the original paper, i.e. the multi-threshold *LQP*, combined with several *loci* of points.	[13]
*Morph*	Strandmark morphological features.	[35]
*LCP*	Multi-scale linear configuration model 2 (radius, neighboring points) configurations: (1,8) and (2,16).	[36]
*MLCP*	The multi-quinary coding version of *LCP*, combined with several *loci* of points.	[13]
*NTLBP*	Multi-scale noise tolerant *LBP* with 2 (radius, neighboring points) configurations: (1,8) and (2,16).	[37]
*DENSE*	Multi-scale densely sampled complete *LBP* histogram with 2 (radius, neighboring points) configurations: (1,8) and (2,16).	[38]
*CLBP*	Completed *LBP* with 2 (radius, neighboring points) configurations: (1,8) and (2,16).	[39]
*CoALBP*	Multi-scale co-occurrence of adjacent *LBP* with radius 1, 2 and 4.	[40]
*RICLBP*	Multi-scale rotation invariant co-occurrence of adjacent *LBP* with radius 1, 2, and 4.	[15]
*WLD*	Weber law descriptor.	[41]
*MLPQ*	Multi-ternary coding version of *LPQ*, with thresholds {0.2, 0.4, 0.6, 0.8, 1}.	[13]
*MLPQens*	Ensemble of *MLPQ* (with a reduced set of thresholds, i.e. {0.2, 0.5, 0.8}, for reducing the computation time) obtained varying several parameters of *LPQ* as the filter size *r =* {3, 5}, the scalar frequency *a =* {0.8, 1.2, 1.6, 2}, and the correlation coefficient between adjacent pixel values *ρ =* {0.75, 1.15, 1.55, 1.95}.	[42]

#### 2.1.2 Preprocessing

One of the aims was to improve the performance of texture descriptors by using a set of different preprocessing methods before feature extraction. When using a given preprocessing approach, a new set of images was produced to be then processed by an ensemble of descriptors. Classification was performed separately for each descriptor using *SVMs*, with both linear and radial basis function kernels, as the base classifier. For each dataset, the best kernel and set of parameters were chosen using a 5-fold cross-validation approach on the training data. *SVMs* were implemented using the tool LibSVM (available at http://www.csie.ntu.edu.tw/~cjlin/libsvm/) and combined by sum rule.

**Fig 1:**
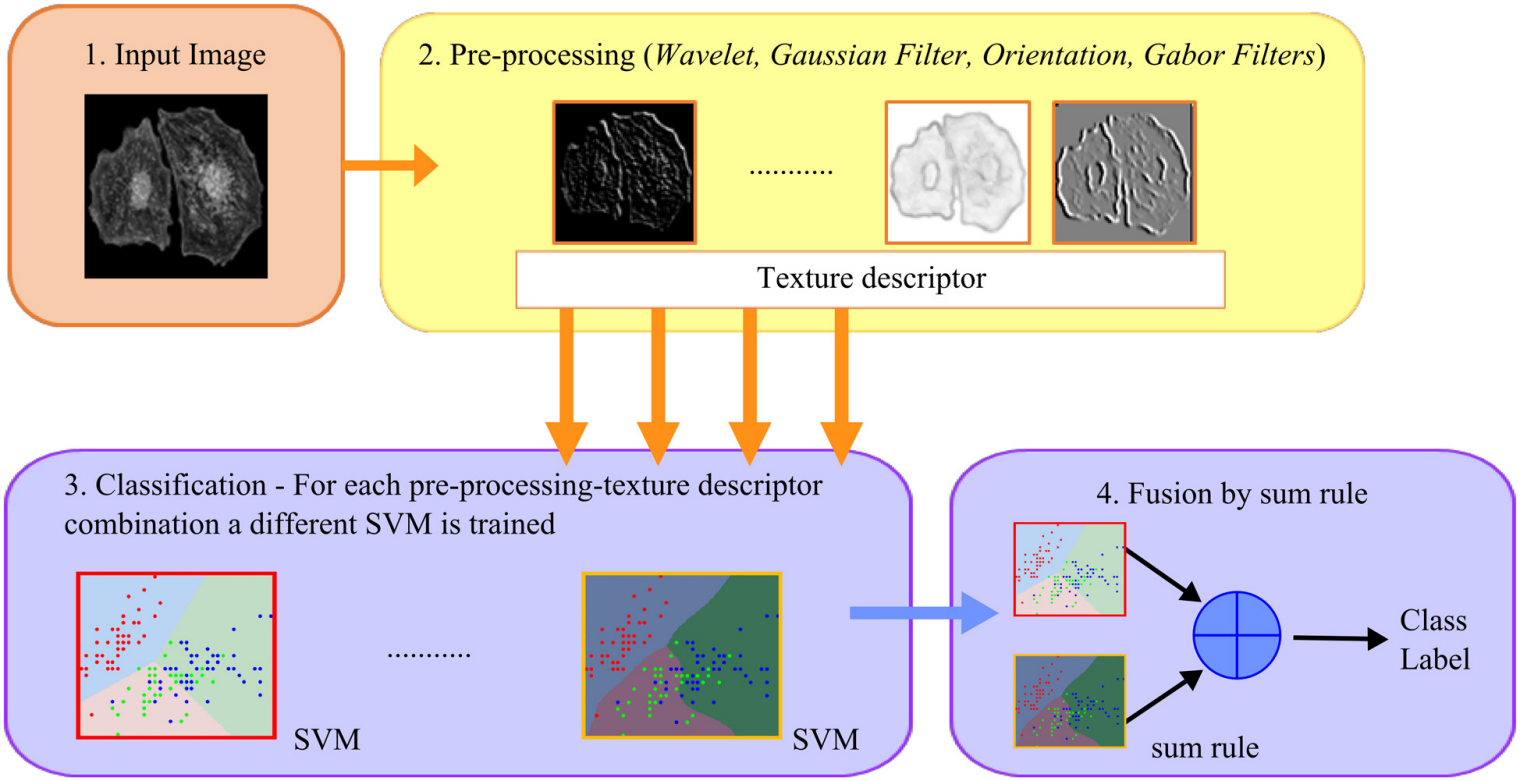
Flowchart of the preprocessing.

The image preprocessing phase included the testing of the following four methods: decomposition by wavelets, multi-resolution by Gaussian filters, orientation image and Gabor filters. Of note, only the training data is used for finding the parameters of the different approaches while the test set is blind. The flowchart of the preprocessing is reported in Fig 1.

Wavelet transform is frequently used in many computer vision problems related with detection and recognition of objects of interest. Wavelet transform [27], to be used for 2D decomposition, requires a 2D scaling function, *φ*(*x*,*y*) and three 2-D wavelets functions, *ψ*^*i*^(*x*,*y*), where *i* represents the three possible intensity variations along horizontal, vertical and diagonal edges *i* = {*H*,*V*,*D*}.

As both functions are separable, the scaled and translated basis functions are defined as:

$$\varphi_{j,m,n}\left(x,y\right)=2^{j/2}\varphi \left(2^{j}x-m,2^{j}y-n\right),$$

$$\psi_{j,m,n}^{i}\left(x,y\right)=2^{j/2}\psi^{i}\left(2^{j}x-m,2^{j}y-n\right),\;i=\left\{H,V,D\right\}.$$

For the three discrete wavelet transform functions (*W*^*H*^,*W*^*V*^ and *W*^*D*^ for horizontal, vertical and diagonal respectively) of a *M* x *N* function *f*(*x*,*y*), the used formulation is:

$$W_{\varphi }\left(j_{0},m,n\right)=\frac{1}{\sqrt{MN}}\Sigma_{x=0}^{M-1}\Sigma_{y=0}^{N-1}f\left(x,y\right)\varphi_{j_{0},m,n}\left(x,y\right),$$

$$W_{\psi }^{i}\left(j,m,n\right)=\frac{1}{\sqrt{MN}}\Sigma_{x=0}^{M-1}\Sigma_{y=0}^{N-1}f\left(x,y\right)\psi_{j,m,n}^{i}\left(x,y\right),\;i=\left\{H,V,D\right\},$$

where *j*_0_ is an arbitrary starting scale and the *W*_*φ*_(*j*_0_,*m*,*n*) coefficients represent an approximation (on the initial scale *j*_0_) of *f*(*x*,*y*). The $W_{\psi }^{i}\left(j,m,n\right)$ coefficients represent the three directional (horizontal, vertical, and diagonal) details for higher scales than *j*_0_.

**Fig 2:**
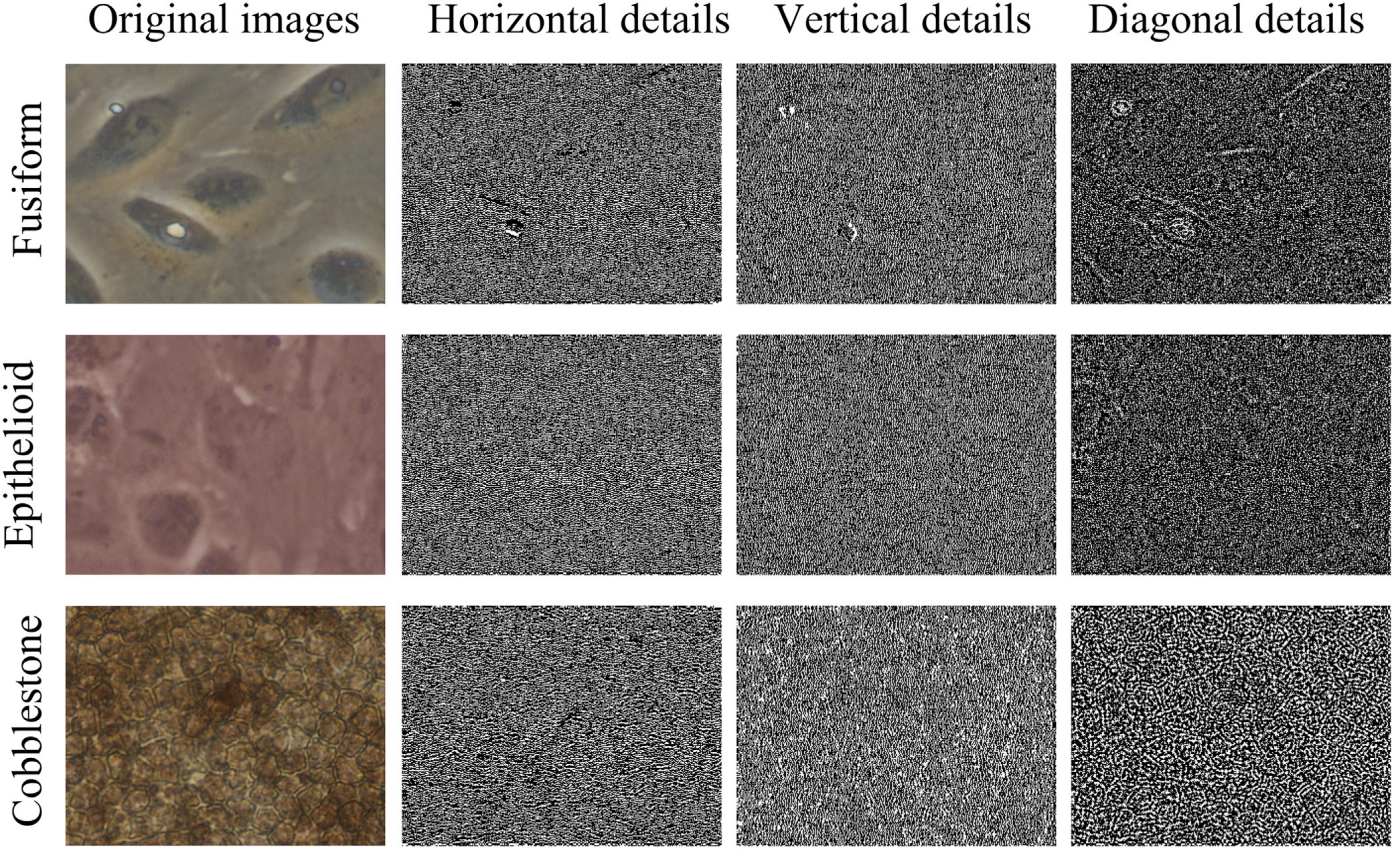
Preprocessing by *Wa*. Rows represent the three classes fusiform, epithelioid and cobblestone. *Left*: original image; *right*: horizontal, vertical and diagonal details.

In our experiments, we used the Daubechies wavelet family (*Wa*) with four vanishing moments. An example of *Wa* processing is shown in Fig 2.

**Fig 3:**
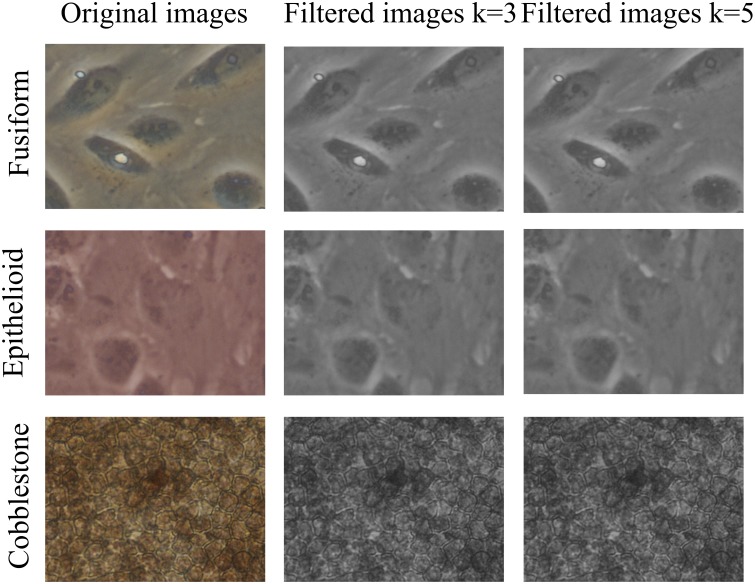
Preprocessing by *MRS*. Rows represent the three classes fusiform, epithelioid and cobblestone. *Left*: original image; *center*: image filtered by a lowpass filter *k* = 3; *right*: image filtered by a lowpass filter *k* = 5.

The second preprocessing technique was a multi-scale approach by means of Gaussian scale-space representation (*MRS*). The original image was filtered to obtain two smoothed versions by using a 2D symmetric Gaussian lowpass filter of size *k* pixels (here we use *k* = 3 and *k* = 5) with standard deviation 1. Illustrative results of *MRS* preprocessing are shown in Fig 3.

The third preprocessing technique was the Orientation image (*OR*). In the Orientation image the image gradient is soft quantized [43] using *d* orientations (here *d* = 3), thereby producing *d* processed images. This method is used to reduce the noise or other forms of degradation.

In detail, *OR* computation is organized in 3 steps:

1. for each pixel, the *p* gradient magnitude *m(p)* and orientation *θ(p)* are computed. *θ(p)* is then discretized over [0, 2π]. The pixel label is a *d*-dimensional vector $\left[{\hat{m}}_{1}\left(p\right),\ldots ,{\hat{m}}_{d}\left(p\right)\right]$ with just one non-null element *i* which is equal to *m(p)* if the discretized *θ(p)* corresponds to the *i*-th bin, i.e.${\hat{m}}_{i}\left(p\right)=m\left(p\right)$. A more refined approach, the soft decomposition of the magnitude, consists in quantizing *m(p)* in two parts to be assigned to the directions of the *p*’s two nearest neighbors. Details about soft decomposition are reported in [43];
2. to include in each pixel the *p* information also from its neighborhood, a local histogram of orientations computed all over the pixels contained into a squared-shape image patch (Cell) centered in *p* and size *w*. At pixel *p* the new feature vector is $\left[{\tilde{m}}_{1}\left(p\right),\ldots ,{\tilde{m}}_{d}\left(p\right)\right]$, where ${\tilde{m}}_{i}\left(p\right)=\underset{p_{j}\epsilon C}{\sum }{\hat{m}}_{i}\left(p_{j}\right)$.
3. finally, a self-similarity measurement computed over *n* cells centered in the *c*_*j*_ pixels surrounding *p* (this topological structure is a circular block of radius *L* centered in *p*): $OR_{L,w,n}^{i}\left(p\right)=\Sigma_{j=1}^{n}f\left({\tilde{m}}_{i}\left(p\right)-{\tilde{m}}_{i}\left(c_{j}\right)\right)2^{j}$, where $f\left(x\right)=\{\begin{array}{cc} 1, & x\geq \tau \\ 0, & otherwise \end{array}$ and *τ* is a threshold slightly greater than 0 to make the mapping stronger in near-uniform regions.

**Fig 4:**
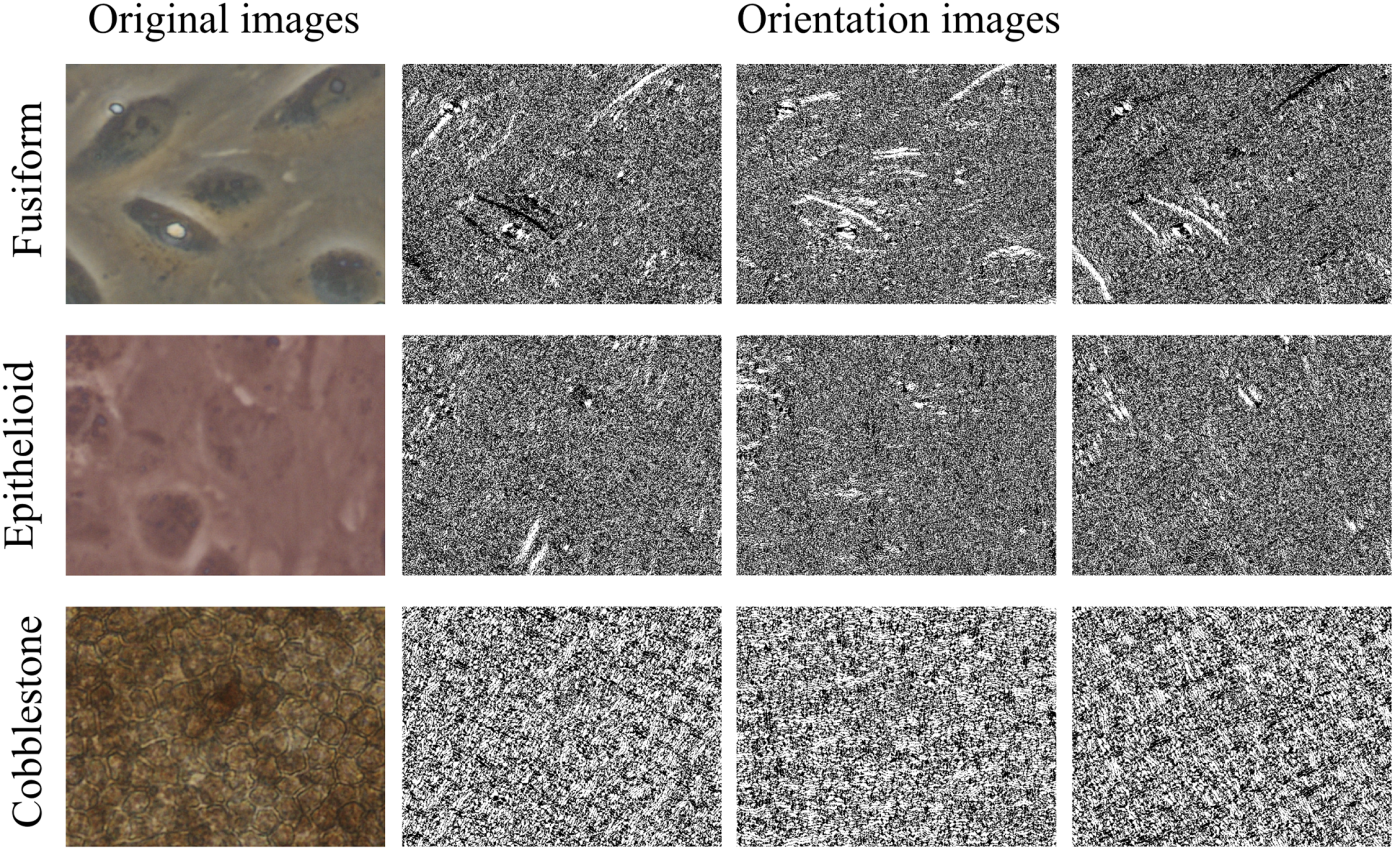
Preprocessing by *OR*. Rows represent the three classes fusiform, epithelioid and cobblestone. *Left*: original image; *right*: the three oriented images.

This produces *d = 3* different orientation images (for details refer to [43]); an example of *OR* preprocessing is shown in Fig 4.

The final preprocessing technique was Gabor filters. A 2D Gabor filter is a Gaussian kernel function modulated by a sinusoidal plane wave that is able to detect frequencies in various scales and directions. Gabor wavelets are derived from the convolution of the input image with a family of Gabor kernels. This family, or bank, of Gabor filters is created by dilating and rotating a specific function. Gabor wavelets approximate, to a certain level, the perception in the primary human visual cortex [28]. In this study four scales {1, 2, 3, 4} and four directions {0°, 45°, 90°, 135°} were implemented, thus 16 images are obtained. Choosing a specific frequency and direction allows creating a map containing the local frequency and orientation information for each pixel in an image.

**Fig 5:**
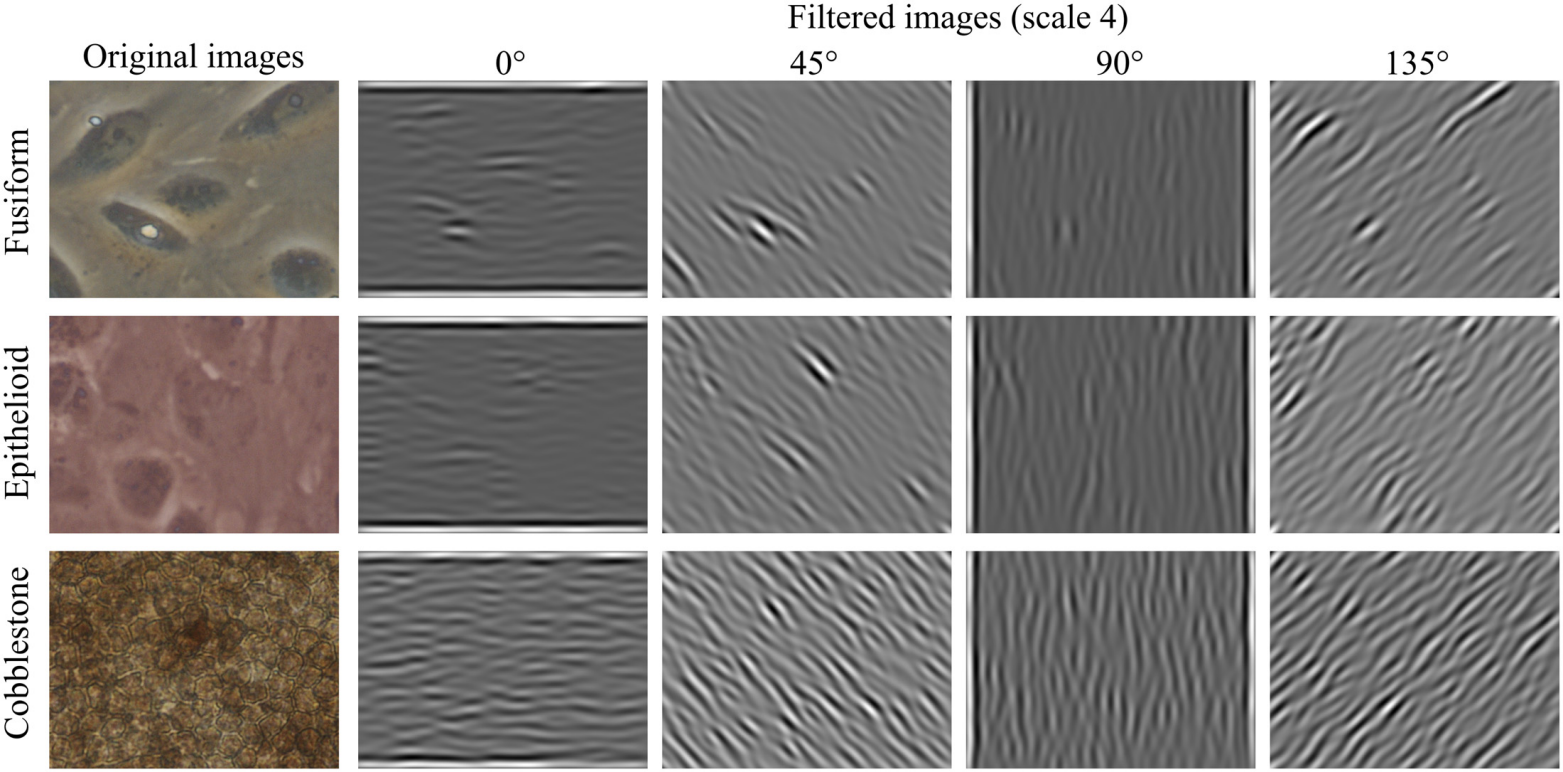
Preprocessing by Gabor filters. Rows represent the three classes fusiform, epithelioid and cobblestone. *Left*: original image; *right*: convolved images at scale 4.

A symmetric Gabor filter has the following general form in the spatial domain:

$$\begin{array}{l} G(x,y,\nu ,\sigma ,\theta )=\text{exp}\left(-\frac{{x^{\prime }}^{2}+{y^{\prime }}^{2}}{2\sigma^{2}}\right)\cdot \text{cos}\left(2\pi \nu x^{\prime }\right)\\ x^{\prime }=x\text{sin}\theta +y\text{cos}\theta \\ y^{\prime }=x\text{cos}\theta -y\text{sin}\theta \end{array}$$

where *ν* is the frequency of the sinusoidal wave, *θ* is the orientation, and *σ* is the standard deviation of the Gaussian envelope [44]. An example of Gabor filter and convolved image is shown in Fig 5.

The various preprocessing techniques, and their ensembles, used in Section 3 are summarized in Table 2.

**Table 2:** Preprocessing approaches.

Acronym	Preprocessing and parameters
*O*	Features extracted only from the original image.
*Wa*	Features extracted from the four images obtained applying the wavelet decomposition to the original image. The four *SVMs* are combined by sum rule.
*WaH*	Features extracted from the images obtained applying a two level decomposition to the original image, as proposed by [14]. The chosen wavelet mother is the same used in *Wa*.
*OR*	Features extracted from the three orientation images obtained from the original image. This ensemble is built by 3 *SVMs*.
*Ga*	The original image is filtered by Gabor filters obtaining 16 images and then features are extracted. The 16 *SVMs* are combined by sum rule
*X+Y*	Fusion by sum rule between preprocessings X and Y.
*Comb*	The fusion by sum rule among *Wa*, *OR* and *Ga*, where preprocessing approaches are applied to the original image and the two images obtained by *MRS*. The final ensemble includes (4+3+16)×3 = 69 *SVMs*.

#### 2.1.3 Region-based descriptors

This idea was mainly inspired by [26], where the edge-based *LBP* variant (*Edge*) is proposed. This bases on the evidence that, when an observer needs to fixate the attention to a particular image, the most likely perceived locations are the ones that present the highest spatial frequency edge information [45].

The *Edge* descriptor is computed as follows:

1. applying *LBP* to an image to obtain the *LBP* image (*LBPI*);
2. detecting the edges in the original image by means of Sobel filter. Two binary maps are created from the edge information: the edge map (*E*, where edge pixels are set to 1 and non-edge pixels to 0), and the non-edge map (*NE*, where edge pixels are set to 0 and non-edge pixels to 1);
3. combining *LBPI* with the *E* and *NE* masks, to obtain two histograms (*H*_*E*_ for edge pixels and *H*_*NE*_ for non-edge pixels), see (Abdesselam, 2013) for details;
4. mounting the final histogram (weighted concatenation):

$$H=\;\left[w_{E}\times H_{E},w_{NE}\times H_{NE}\right],w_{e}>w_{NE}$$

where *w*_*E*_ and *w*_*NE*_ represent the empirically determined weight that express the greater relevance of edge regions in capturing the viewer’s visual attention;

Unlike in [26], in this study the two histograms, *H*_*E*_ and *H*_*NE*_, were not combined into one feature vector but they were used separately to train two different *SVM*s that are then combined by sum rule.

**Fig 6:**
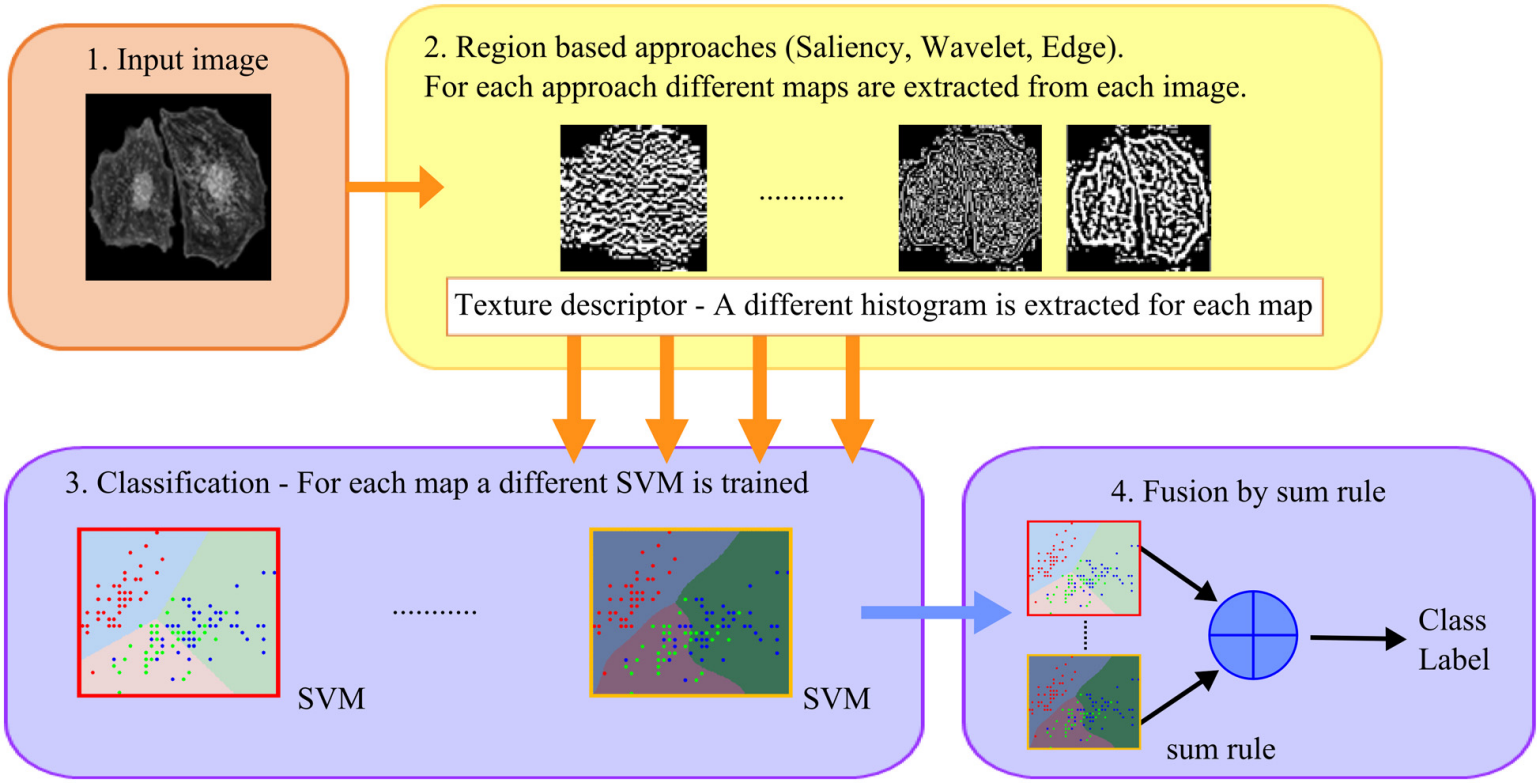
Flowchart of the region-based approach.

Two methods for extracting the two maps were tested: the former based on saliency and the latter on wavelet decomposition. It should be noted that for both approaches, as in *Edge*, the descriptor was extracted initially from the original image and the two regions were used only to calculate the two histograms. The flowchart of the region-based approach is reported in Fig 6.

In detail:

1. the chosen descriptors (namely *LBP*, *LTP*, *LPQ*, *RICLBP* and *WLD*) were applied to the texture image to get the labeled image *DescI*;
2. two maps, *Map*^*+*^ and *Map*^*-*^, were computed according to saliency or wavelet (details in the next sections);
3. two histograms, *H*^*+*^ and *H*^*-*^, were computed by combining *DescI* with *Map*^*+*^ and *Map*^*-*^, respectively;
4. *H*^*+*^ and *H*^*-*^ were used to train two different *SVM*s that were then combined by sum rule.

*Saliency*: We used the method proposed in [46] to extract a saliency map from the image. Given an image **x**, the signature is defined as

ImageSignature(x)=sign(DCT(x)),

where *sign()* represents the sign operator and *DCT()* is the Discrete Cosine Transform. Hou *et al*. [46] demonstrated analytically and experimentally that the support of the foreground of an image can be approximated by the reconstructed image $\bar{x}$, obtained by transforming back to the spatial domain the image signature, as follows:

$$\bar{x}=IDCT\left(ImageSignature\left(x\right)\right).$$

For images whose foreground is evident compared to their background, the saliency map ***m*** is defined as

$$m=g*\left(\bar{x}\;o\;\bar{x}\right),$$

where *g* is a Gaussian kernel aimed to blur the noise induced by the sign quantization and *o* is the entrywise matrix product operator. To build the saliency map the standard deviation of the Gaussian kernel was set to 2.

For each image two regions were extracted. The first contained the pixels with saliency higher and the latter contained the pixels with saliency lower than a prefixed threshold. To build the saliency map two different thresholds were applied: 0.5 and 0.7. Hence, for each image, two saliency maps and four histograms were extracted.

*Wavelet*: The wavelet decomposition (see section 2.1.2) used four wavelets, and the horizontal, vertical and diagonal coefficients matrices were considered. These matrices were resized to the size of the original image and then the mean value of each image was calculated. Each image was divided in two regions whose pixels were respectively greater and smaller than the mean value.

The region-based methods, as well as the baselines, are summarized in the following Table 3.

**Table 3:** Region-based methods and baselines (BAS) used for comparison.

	Acronym	Region-based method
BAS	*O*	Standard texture descriptor applied to the original image.
	*DoG*	The Difference of Gaussians approach proposed in [25].
Region-based	*Saliency + Wavelet*	Fusion by sum rule between *Saliency* and *Wavelet;*
	*All*	The fusion by sum rule among *Saliency*, *Edge* and *Wavelet*;
	*All* + *Comb*	The descriptors of *All* are extracted from the images obtained using *Wa* and *OR*, where the preprocessing approaches are applied to the original image and the two images obtained by *MRS*. Due to computation time *Ga* was not tested.

#### 2.1.4 Binarized Statistical Image Features

The *Bsif* descriptor assigns an n-bit label to each pixel of a given image by exploiting a set of *n* linear filters. Given a neighborhood of *l x l* pixels and a set of *n* linear filters of the same size, the n-bit label to be assigned to the central pixel of the neighborhood is obtained by binarizing

s=Wx

where ***x*** is the *l*^*2*^ *x 1* vector notation of the *l x l* neighborhood and ***W*** is a *n x l*^*2*^ matrix representing the stack of the vector notations of the filters. In detail, the *i*-th digit of *s* is a function of the *i*-th linear filter ***w***_***i***_ and it is expressed as

$$s_{i}=w_{i}^{T}x,$$

thus each bit of the *Bsif* code can be obtained as

$$b_{i}=\{\begin{array}{cc} 1, & if\;s_{i}>0\\ 0, & if\;s_{i}\leq 0 \end{array}.$$

The set of filters ***w***_***i***_ is estimated by maximizing, through independent component analysis, the statistical independence of the filter responses ***s***_***i***_ on a set of patches from natural images. In the original *Bsif*, the binarized feature *b*_*i*_, was obtained by setting *b*_*i*_ = 1 if s_i_ > *th* and *b*_*i*_ = 0 where *th* = 0. We improved the stand-alone version of *Bsif* by combining different *Bsif* in two ensembles, *Size_Bsif* and *Full_Bsif*. *Size_Bsif* was obtained by varying the filter *size* = {3, 5, 7, 9, 11} (i.e. we use 5 different filters). The second ensemble, *Full_Bsif*, was derived from *Size_Bsif* by varying also the threshold *th* used to binarize the image. In detail, we used the following thresholds *th* = {-9, -6, -3, 0, 3, 6, 9} for each different size of the filter and, the 35 *SVM*s trained with these *Bsif*-based descriptors (for each couple of size and threshold a different *SVM* is trained) were combined by sum rule.

#### 2.1.5 Multi-quinary coding

Variants of the original *LBP* descriptor were proposed, based on modifications on the binarizing function *s(x)*, originally defined in [21] as:

$$s\left(x\right)=\{\begin{array}{ll} 1, & x\geq 0\\ 0, & otherwise \end{array},$$

where *x* = *q*_*p*_ −*q*_*c*_, *q*_*c*_ represents the central pixel in the neighborhood and *q*_*p*_ each of the surrounding pixels.

In [22], *LTP* was defined by encoding the same difference *x* with 3 values, by means of the threshold τ:

$$s\left(x\right)=\{\begin{array}{rr} 1, & x\geq \tau \\ 0, & -\tau \leq x<\tau \\ -1, & otherwise \end{array}$$

In [13], this approach was extended to *LQP* by introducing two thresholds τ_1_ and τ_2_ (τ_1<_τ_2_), thus getting the quinary coding:

$$s\left(x\right)=\{\begin{array}{rr} 2, & x\geq \tau_{2}\\ 1, & \tau_{1}\leq x<\tau_{2}\\ 0, & -\tau_{1}\leq x<\tau_{1}\\ -1 & \tau_{2}\leq x<-\tau_{1}\\ -2 & otherwise \end{array}$$

These variants of the binary coding allow a lower sensitivity to noise, especially in near-uniform regions, and a higher level of granularity that allows catching more textural features with respect to the original version. However, to compensate the increased verbosity of the ternary and quinary codings, the ternary patterns are split into one positive and one negative binary patterns, according to the sign of its components, while the quinary patterns are split into four binary patterns, according to *b*_*c*_*(d)*:

$$b_{c}\left(d\right)=\{\begin{array}{ll} 1, & d=c\\ 0, & otherwise \end{array}$$

where *c*∈{-2, -1, 1, 2} and *d* represents a single digit of the quinary pattern. For instance, the first binary pattern results from *c* = 2, the second one from *c* = 1 and so on for *c* = -1 and *c* = -2.

After computing one histogram for each binary pattern, the six partial histograms (two for *LTP* and four for *LQP*) are concatenated into a final histogram.

Moreover, in [13], a multi-threshold version of *LQP*, namely multi-threshold *LQP* (*MLQP*) was proposed, using a set of 25 couples of thresholds (*τ*_*1*_ = {1,3,5,7,9} and *τ*_*2*_ = {τ_1_+2, τ_1_+4,…, τ_1_+11}) and combining the 25 *SVM*s trained with the histograms. Usually in *LBP*, and in its variants, a circular neighborhood allows obtaining a rotation invariant descriptor. However, in some problems, anisotropy is an important source of information. To use the anisotropic structural information, several neighborhood shapes (such as parabola, ellipse and hyperbole) were used in [12] (Table 4).

**Table 4:** *Loci* of points defining the different neighborhood topologies. For each geometric *locus* defined in [12], its formal definition and parameters are reported.

*Locus*	Definition	Parameters
Circle	*x*^2^ + *y*^2^ = *r*^2^	*r*: radius.
Ellipse	x2a2+y2b2=1	*a*: semi-major axis; *b*: semi-minor axis.
Parabola	y=−x2c+2c	*c*: vertex—focus distance.
Hyperbola	x2a2−y2b2=1	*a*: semi-major axis; *b*: semi-minor axis.
Spiral	*r* = *a* + *b*θ	*a*: turns the spiral; *b*: sets the inter-turnings distance; *r*, *θ*: polar coordinates.

In *MLQP*, the threshold selection is a critical task: in [13], we set the thresholds manually to get good performance in studied datasets. The proposed thresholds were stable enough also in the RPE classification problem. The performance of *MLQP* was enhanced by building a large set of *LQP* coupling the set of thresholds with the geometric *loci* presented in [12] and summarized in Table 4.

**Fig 7:**
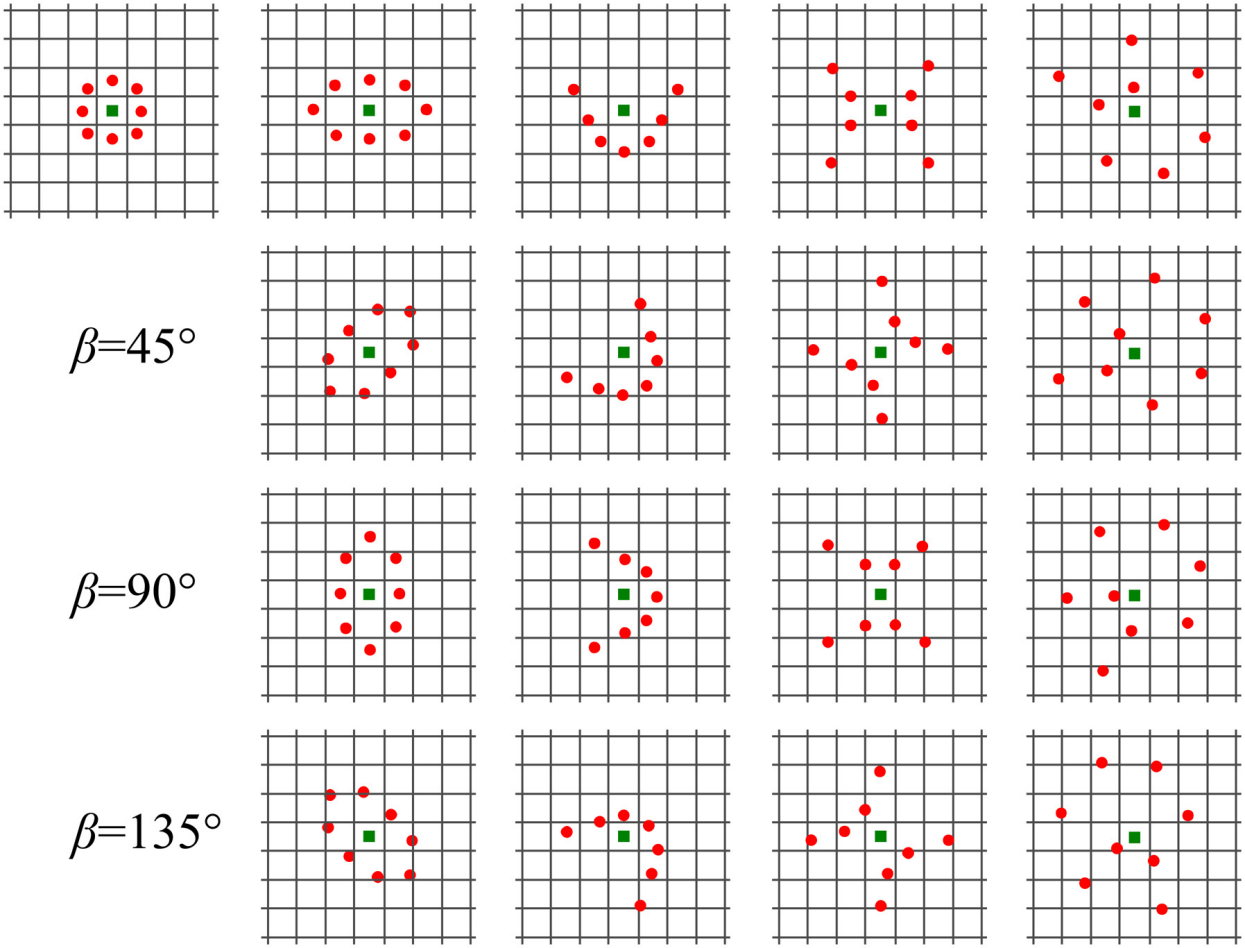
The different neighborhood topologies. From *left* to *right* in line 1: circle, ellipse, parabola, hyperbola and spiral. We represented the central pixel of the neighborhood (green) and the points forming the neighborhood (red). In line 2, 3 and 4 the different rotation angles *β* are represented.

**Fig 8:**
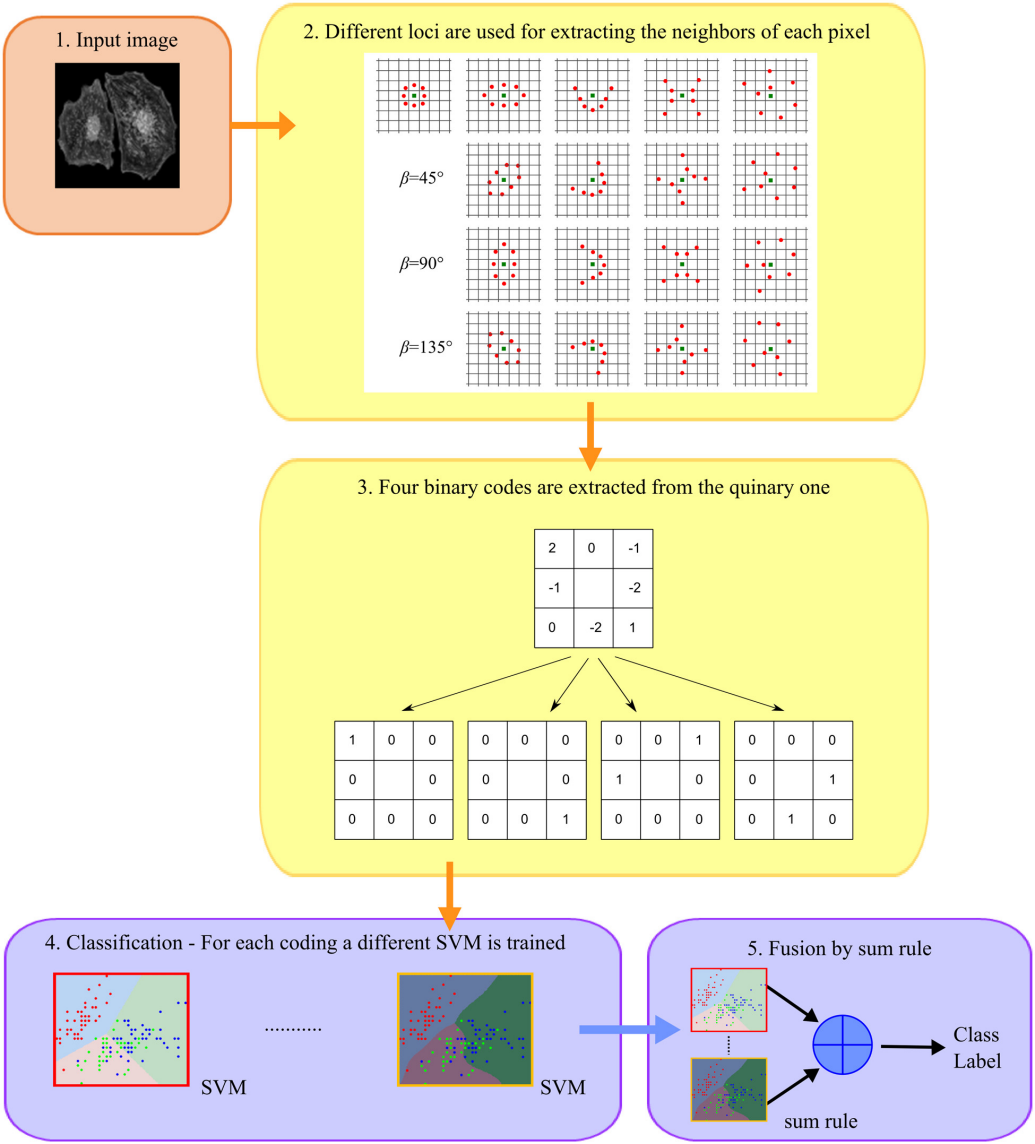
Flowchart of the quinary coding and usage of the geometric *loci*.

All the *loci* of points (with the exception of the circle) were rotated by *β* = {0°, 45°, 90°, 135°} to catch the anisotropic structural information according to different orientations as in Fig 7. The flowchart of the quinary coding and the use of the geometric *loci* is reported in Fig 8.

Afterwards, the Sequential Forward Floating Selection (*SFFS*) was applied, using the training data for selecting only a single subset of the *MLQP* descriptors.

*SFFS* and its predecessor Sequential Forward Selection (*SFS*) are top-down searches that sequentially select a subset of features from the original set of candidates in order find an optimal subset.

Starting from the empty subset *S*_*0*_, *SFS* sequentially adds the *k*-th feature, maximizing the objective function when combined with the subset *S*_*k-1*_ of the previously selected *k-1* features, thus getting the current subset *S*_*k*_. However, the main drawback is that the selected features cannot be reevaluated and discarded after the addition of a new feature.

*SFFS* [47] improves *SFS* by carrying out backward steps after the inclusion of a new feature as long as the objective function rises. For instance, after the *k*-th step forward, i.e. the selection of the *k*-th feature, each feature in *S*_*k*_ is removed from the subset to get a smaller subset $S_{k-1}^{\prime }$ whose performance is compared with *S*_*k−1*_’s. If $S_{k-1}^{\prime }$ results in a greater objective function than *S*_*k−1*_, then it replaces *S*_*k−1*_.

We used *SFFS* as a feature selection method, where each feature was assigned a couple of thresholds and a geometric *locus*, to find the most useful (thresholds, *locus*) sets for *MLQP*. Therefore, we selected a set of *MLQP* descriptors, where each descriptor was used to train a given *SVM*: the objective function of *SFFS* was the maximization of the area under the *ROC* curve (obtained combining by sum rule the set of *SVM*s) using an internal 10-fold cross validation in the training data. The same procedures (thresholds, geometric *loci* and feature selection) were used also to extend the Local Configuration Pattern (*LCP*) descriptor to its multi-threshold quinary version *MLCP*.

#### 2.1.6 Color descriptors

For the RPE dataset only (Section 2.2) we used an additional set of descriptors, not based on texture, but on colors (COLORS). COLORS consists in the concatenation of statistics computed on the three channels of a color image: mean, homogeneity, standard deviation, third, fourth and fifth moments and the marginal histograms (8 bins for each channel) [48,49].

### 2.2 The RPE dataset

#### 2.2.1 Cell culture

Two hESC lines (Regea 08/023; 46, XY, Regea 08/017; 46,XX) [50] and one human induced pluripotent stem cell (hiPSC) line, (UTA.04511.WTS 46, XY) [51] were used for this study. Cell lines were cultured on top of mitomycin-treated (10 μg/ml,Sigma-Aldrich) (i.e. mitotically inactivated) human foreskin fibroblasts feeder cells (CRL-2429TM, ATCC, Manassas, VA, USA). The undifferentiated cells were cultured similarly as in Sorkio *et al*. [52] and after one week of culture the differentiation was induced by reducing the KO-SR concentration to 15%, removing the bFGF and commencing the floating culture as previously described in Vaajasaari *et al*. [53]. Floating aggregates were fed thrice a week and grown for 70–195 days. The pigmented areas of floating aggregates were manually dissected, dissociated with 1x Trypsin-EDTA and replated on collagen IV from human placenta (5 μg/cm^2^, Sigma-Aldrich). Adherently cultured cells were imaged for the fusiform morphology after 8 days (range 6–9 days), for the epithelioid morphology after 9 days (range 8–9) and for the cobblestone morphology after 19 days (range 17–24) of culturing.

#### 2.2.2 Ethical issues

The National Authority for Medicolegal Affairs Finland has approved our research with human embryos (Decision number 1426/32/300/05). We also have a supportive statement from the local ethics committee of the Pirkanmaa hospital district Finland to derive and expand hESC lines for research purposes (R05116). Local ethics committee of the Pirkanmaa Hospital District has given a supportive statement to generate iPSC lines for research purposes (R11028), and use them to ophthalmic research (R14023). No new hESC or hiPSC lines were generated in this study.

#### 2.2.3 Image acquisition

The cell culture images were acquired for analysis with the same settings (25–125 ms exposure time, 2560 x 1920 pixels, dynamic contrast and autowhite balance) from cell cultures using a Nikon Eclipse TE200S phase-contrast microscope (Nikon Instruments Europe B.V., Amstelveen, Netherlands) with the 20x objective and Ph1 phase contrast. Cell imaging parameters are described in Table 5.

**Table 5:** Image acquisition parameters.

Acquisition parameter	Value
Quality	2560 x 1920 pixels
Mode	Manual exposure
Exposure	25–125 ms
AE compensation	0
Gain	1.2 x
Contrast	Dynamic

#### 2.2.4 Building the RPE dataset

**Fig 9:**
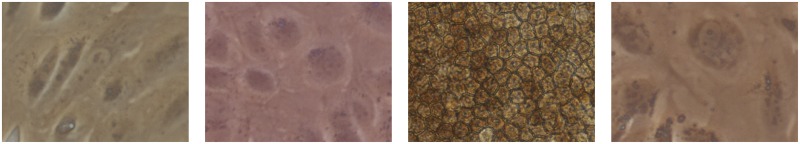
Illustrative images of the RPE maturation stages (classes). From *left* to *right*: fusiform, epithelioid, cobblestone and mixed (Fusiform and Epithelioid).

Each acquired image was divided into 16 subwindows which were manually labeled into 4 classes by two trained operators; samples particularly difficult to be labeled were inspected by a specialist. Subwindows containing clutters, out-of-focus elements or just background were discarded. The criteria of inclusion and examples are shown for each class in Table 6 and in Fig 9.

**Table 6:** Class properties used for building the ground truth.

Class (# subwindows)	Features
Fusiform (216)	Fuse shaped cell contours and nucleus
	Separated cells
Epithelioid (547)	Globular shaped cell contours and nucleus
	More packed
Cobblestone (949)	Well defined cell contours and cell wall
	Hexagonal shape
	Homogeneous cytoplasm
	Tightly packed
Mixed (150)	Two or more of aforementioned classes.

In Fig 9, the four RPE classes are represented from left to right: the fusiform cell type, the epithelioid with its characteristic globular shape, the final maturation stage cobblestone and a mixed class example with an epithelioid cell in the middle of the image and, in the left side, a cluster of fusiform cells. The final dataset includes a total of 1862 subwindows: the number of subwindows per class is reported in Table 6. Before using the RPE images, they were converted to gray scale by means of the standard MATLAB (The MathWorks, Inc., Natick, Massachusetts, United States) function *rgb2gray*.

### 2.3 Validation in other datasets

For validating some of the proposed variants of texture descriptors and the system for RPE classification, we ran several comparisons also in other datasets. As testing protocol, we used the 5-fold cross validation, except for the VIR dataset for which the 10-fold validation protocol was provided by the original author.

The following datasets were used:

- **PAP**: this dataset [54] contains 917 images unevenly distributed among 7 classes of cells, acquired during Pap tests for the diagnosis of cervical cancer. The dataset is available upon request to Loris Nanni [nanni@dei.unipd.it];
- **VIR**: this dataset [55] contains 1500 images, evenly divided into 10 classes, of viruses extracted using negative stain transmission electron microscopy. The 10-fold validation protocol shared by the authors was used. The mask for background subtraction was not used and the features were extracted from the whole images. The dataset is available at http://www.cb.uu.se/~gustaf/virustexture/;
- **HI**: this Histopathology dataset [56] is composed of 2828 images from different organs, unevenly distributed among 4 classes, representative of the four fundamental tissues (connective, epithelial, muscular, and nervous). The dataset is available upon request to Loris Nanni [nanni@dei.unipd.it];
- **BR**: this dataset [57] is a subset of the digital database for screening mammography [58] and contains 1394 images of breast tissue, 810 control, 273 malignant and 311 benign breast cancers. The dataset is available upon request to Geraldo Braz Junior [ge.braz@gmail.com];
- **PR**: this dataset, reported in [59] contains 329 proteins, divided into *DNA*-binding (118 samples) and non-*DNA*-binding (231 samples). From the 3D tertiary structure of each protein, its 2D distance matrix was computed (considering only atoms that belong to the protein backbone) and used to extract texture features. The dataset is available upon request to Loris Nanni [nanni@dei.unipd.it];
- **CHO**: this cell dataset [24] contains 327 fluorescent microscopy images of Chinese Hamster Ovary cells and distributed into 5 classes. The dataset is available at http://ome.grc.nia.nih.gov/iicbu2008/hela/index.html#cho;
- **HeLa**: the 2D HeLa dataset [23] consists in 862 single cell images, divided into 10 staining classes, from fluorescence microscope acquisitions on HeLa cells. The dataset is available at http://ome.grc.nia.nih.gov/iicbu2008/hela/index.html;
- **LE**: the LOCATE ENDOGENOUS mouse sub-cellular organelles dataset [60] contains 502 images, unevenly distributed among 10 classes, of endogenous proteins or features of specific organelles. The dataset is available at http://locate.imb.uq.edu.au/;
- **LT**: the LOCATE TRANSFECTED mouse sub-cellular organelles dataset [60] contains 553 images, unevenly distributed in 11 classes, of fluorescence- or epitope-tagged protein transiently expressed in specific organelles. The dataset is available at http://locate.imb.uq.edu.au/;
- **RNAi**: this dataset contains 200 fluorescence microscopy images, evenly distributed among 10 classes, of fly cells subjected to a set of gene-knockdowns using *RNAi* and stained with *DAPI* to visualize their nuclei. The dataset is available at http://ome.grc.nia.nih.gov/iicbu2008/rnai/index.html.

## Results and Discussion

### 3.1 Experimental results for the RPE dataset

The chosen performance indicator was the area under the *ROC* curve (*AUC*) as it is more reliable than accuracy. *AUC* allows summarizing in one scalar value the *ROC* curve. In multi-class problems the *one-versus-all* approach was used: each of the *m* classes was considered as “positive” and the remaining *m-1* classes as “negative”, thus obtaining *m* partial *AUC*s. Finally, the global *AUC* was computed as an average of the partial *AUC*s.

As testing protocol a 10-fold cross validation was used. Of note, the 10-fold was applied at image level, so all the sub-windows of a given image belonged or to the training set or to the test set.

Of note, when referring to a texture descriptor combined with preprocessing/region-based approaches, we use the notation *preprocessing(descriptor)*. For example, *Comb(RICLBP)* means *RICLBP* combined with the various preprocessing *Wa*, *OR* and *Ga* applied to the original image and the two images obtained by *MRS* (see Table 2).

In the first test, reported in Table 7 several texture descriptors and five different ensembles (the last five rows) were compared. The methods named A+B are the fusions by sum rule between the methods A and B. The *LBP*-based approaches use uniform bins, except in presence of the suffix -*ri*, representing the rotation invariant bins. From the results reported in Table 7, it is clear that the best performances were obtained using descriptor ensembles. The best performance was reached by the ensemble *RICLBP*+*MLPQens*+*MLCP*, while the best stand-alone approach was *RICLBP*. We used an already published method [61] for assessing the difference between two approaches in the same dataset. MLCP, i.e. the multi-threshold quinary ensemble of LCP built according to [13] (see section 2.1.5), outperforms RICLBP with a probability of 80% using a one sided test of significance with p = 0.05. However, the proposed ensembles *RICLBP* + *MLPQens* + *MLCP* and *Comb(RICLBP)* + *Full_Bsif* + *MLPQens* + *MLCP* outperform each stand-alone approach with a probability of 95%, using a one sided test of significance with *p*-value of 0.05.

**Table 7:** Performance (*AUC*) comparison among different texture descriptors.

Descriptor	*AUC*
*LBP-HF*	*76*.*72*
*LPQ*	*77*.*89*
*HoG*	*75*.*53*
*LBP*	*77*.*89*
*LBPri*	*74*.*27*
*LTP*	*79*.*70*
*LTPri*	*75*.*91*
*LCP*	*76*.*87*
*LCPri*	*78*.*11*
*NTLBP*	*74*.*79*
*DENSE*	*80*.*33*
*DENSEri*	*77*.*02*
*Morph*	*79*.*04*
*CLBP*	*78*.*50*
*CoALBP*	*78*.*37*
*RICLBP*	*80*.*34*
*WLD*	*79*.*08*
*MLPQ*	*81*.*82*
*MLPQens*	*82*.*66*
*MLQP*	*81*.*22*
*MLCP*	*83*.*03*
*RICLBP*+*WLD*	*83*.*02*
*RICLBP*+*WLD*+*MLQP*	*83*.*63*
*RICLBP*+*WLD*+*MLCP*	*84*.*34*
*RICLBP*+*MLQP*+*MLCP*	*84*.*16*
*RICLBP*+*MLPQens*+*MLCP*	***84*.*60***

We tested also the effectiveness of COLORS, obtaining an *AUC* of 89.10%.

In Table 8 different approaches based on *Bsif* were compared. The method named *Baseline* represents the standard stand-alone *Bsif* with size = 7. Moreover, *Full_Bsif* was coupled with the best previous ensemble (i.e. *RICLBP*+*MLPQens*+*MLCP*) increasing slightly the performance.

**Table 8:** Performance (*AUC*) comparison among different *Bsif*-based approaches.

Descriptor	*AUC*
*Baseline*	78.50
*Size_Bsif*	79.44
*Full_Bsif*	82.79
*RICLBP*+*MLPQens*+*MLCP*	84.60
*RICLBP*+*MLPQens*+*MLCP*+*Full_Bsif*	85.03

The performance of the best descriptors was presented in Table 9, coupled with the preprocessing approaches detailed in section 2.2 and Table 2.

**Table 9:** Performance (*AUC*) obtained coupling the best texture descriptors with different preprocessing methods.

	Preprocessing							
Descriptor	*O*	*Wa*	*OR*	*Ga*	*WaH*	*Wa+OR*	*Wa+OR+Ga*	*Comb*
*LBP*	77.89	77.05	79.16	77.89	76.84	78.85	79.99	**81.00**
*LTP*	79.70	77.47	76.03	79.13	78.90	78.17	80.82	**82.08**
*LCP*	76.87	77.87	78.57	75.39	77.69	79.31	79.38	**80.77**
*LPQ*	77.89	78.31	80.09	76.67	78.61	80.32	80.45	**80.88**
*RICLBP*	80.34	78.30	79.79	80.11	78.28	80.60	81.77	**82.62**
*WLD*	79.08	79.17	80.30	78.16	78.76	80.92	**81.56**	81.29

Notice that all the preprocessing approaches are coupled with only stand-alone texture descriptors due to the high computation time. The performances of all the descriptors were improved when the features were extracted from *Comb*, comparing with the performance obtained by *O*.

The region-based methods, proposed in section 2.3 and Table 3, were compared in Table 10.

**Table 10:** Performances (*AUC*) of the region-based approaches.

	Region-based method							
Descriptor	*O*	*Edge*	*DoG*	*Saliency*	*Wavelet*	*Saliency+Wavelet*	*All*	*All+Comb*
*LBP*	77.89	79.95	79.23	79.21	80.36	80.63	**80.78**	80.08
*LTP*	79.70	80.01	79.48	79.93	80.78	81.24	**81.34**	80.85
*LPQ*	77.89	78.74	79.80	79.07	79.44	79.71	79.74	**80.12**
*RICLBP*	80.34	81.01	81.29	81.66	81.83	82.33	**82.27**	81.90
*WLD*	79.08	79.98	79.67	78.01	79.97	79.70	80.09	**81.33**

It is clear that a descriptor applied to a set of processed images drastically outperformed the same descriptor obtained using only the original image. However, only some methods’ performances benefited from the different preprocessing methods. The baseline multi-quinary approach (i.e. all the descriptors extracted from the circle neighborhood) and the effect of *SFFS* are reported in Table 11. *SFFS* supervised selection improved *MLQP* but had no positive effect on *MLCP*.

**Table 11:** Performance (*AUC*) of the multi-quinary approaches.

	Supervision	
Descriptor	*no SFFS*	*SFFS*
*MLQP*	81.22	81.71
*MLCP*	83.03	81.15

The final ensemble was created by sum rule among *Comb(RICLBP)*, *Full_Bsif*, *MLPQens* and *MLCP*. It obtained an *AUC* of 86.49%. As shown in Table 6, the RPE dataset was unbalanced towards the cobblestone class, consequently a single 10-fold cross validation risks to create a training set not representative of the classes with fewer subwindows. Therefore we ran a higher-performance but more computationally demanding protocol: the leave-one-image-out, consisting in leaving out one full image (i.e. the image divided into the 16 subwindows) for each round. We tested such protocol on our best ensemble *Comb(RICLBP)* + *MLPQens* + *MLCP* + *Full_Bsif*, whose performance increased from 86.49% to 91.98%. A further improvement was obtained by the fusion by sum rule between such ensemble and COLORS, obtaining an *AUC* of 95.00%.

### 3.2 Results with other datasets

Afterwards, the proposed ensemble of *Bsif*, the preprocessing applied before feature extraction and the region-based descriptors were validated with the other datasets.

Other tests, e.g. coupling *MLCP* with the selection of the geometric *loci*, were not performed due to their huge computational time.

As in the previous tests, the performance indicator was the *AUC*. Moreover, the experiments were statistically validated with the Wilcoxon signed rank test and the Bonferroni-Holm method.

The performances of *Bsif* and of standard texture descriptors, as baseline, were compared in Table 12. The three best baseline methods were *CLBP*, *RICLBP* and *LTP*. *LTP* outperformed all the other baseline approaches (except *RICLBP* and *CLBP*) with a *p*-value of 0.05. Furthermore, there was no difference between the performance of *RICLBP* and *LTP*. Moreover, *Full_Bsif* outperformed both *Bsif*, *Size_Bsif* and all the baseline approaches, including *LTP* with a *p*-value of 0.05.

**Table 12:** Comparison of the performance (*AUC*) of standard texture descriptors and *Bsif* coding.

	Descriptor										
Dataset	*LPQ*	*LBP*	*LCP*	*MLCP*	*CLBP*	*RICLBP*	*WLD*	*LTP*^1^	*Bsif*	*Size_Bsif*	*Full_Bsif*
PAP	90.2	90.0	77.7	81.2	**92.5**	91.8	80.2	91.4	87.1	90.1	**91.4**
VIR	94.9	92.0	87.4	89.2	94.8	**97.6**	86.3	93.5	91.2	96.3	**97.0**
CHO	99.2	99.4	98.8	99.1	**99.9**	99.2	**99.9**	**99.9**	99.3	99.6	**99.9**
HI	92.0	90.6	83.5	86.2	91.8	**92.8**	88.7	91.6	91.0	93.1	**94.0**
BR	95.7	93.6	93.1	95.2	95.8	92.8	92.5	**96.9**	94.8	95.4	**96.7**
PR	86.2	81.0	18.3	20.2	86.6	88.6	86.5	**89.7**	89.2	**93.2**	91.9
HeLa	97.2	98.0	84.4	88.2	98.1	97.3	94.1	**98.6**	97.2	98.3	**99.2**
LE	97.6	98.6	86.8	87.6	98.5	99.0	97.9	**99.5**	98.7	99.5	**99.8**
LT	97.7	98.5	96.7	97.2	98.5	98.7	98.8	**99.3**	98.6	99.2	**99.8**
RNAi	95.2	94.7	94.5	95.3	95.0	96.6	**97.4**	97.0	93.5	96.1	**98.2**
Average	94.6	93.6	82.2	83.9	95.1	95.4	92.2	**95.7**	94.1	96.1	**96.8**

^1^in this work we used the normalized histograms, while in [13] the non-normalized histograms. Therefore, for the same dataset, and for the same testing protocol, different results were reported.

In order to avoid reporting a massive amount of results, in the following we summarized only the results from the best performing baseline descriptors, i.e. *RICLBP*, *LPQ* and *LTP*.

The effect of preprocessing on the additional datasets was reported in Table 13. We also tested the ensemble of preprocessing *O+Wa+OR*, i.e. the sum rule among the preprocessing approaches *O*, *Wa* and *OR* applied to the original image and to the two images obtained by *MRS*. We can conclude that, among the stand-alone preprocessing, *O* is the best one and that the best approach is the ensemble *O+Wa+OR*, which outperformed the baseline approach *O* with a *p*-value of 0.05.

**Table 13:** *AUC* obtained using the preprocessing approaches and *LTP*, *RICLBP* and *LPQ*.

		Preprocessing					
Descriptor	Dataset	*O*	*Wa*	*OR*	*Ga*	*Comb*	*O+Wa+OR*
***LTP***	PAP	**91.4**	88.4	88.8	76.3	87.0	90.1
	VIR	93.5	91.1	93.6	85.8	95.8	**96.0**
	CHO	**99.9**	99.3	**99.9**	99.7	**99.9**	**99.9**
	HI	91.6	90.9	92.5	92.5	**94.6**	93.9
	BR	96.9	97.2	**98.2**	83.6	95.8	96.9
	PR	89.7	79.1	90.6	88.3	91.4	**92.5**
	HeLa	98.6	96.1	97.2	96.1	98.2	**98.8**
	LE	99.5	99.1	99.7	98.3	**99.8**	**99.8**
	LT	99.3	98.8	99.3	98.4	**99.8**	**99.8**
	RNAi	97.0	93.2	96.1	93.3	96.9	**97.7**
	Average	95.7	93.3	95.6	91.2	95.9	**96.5**
***RICLBP***	PAP	**91.8**	84.1	84.8	76.1	87.5	91.5
	VIR	97.6	93.2	92.8	87.0	95.7	**97.9**
	CHO	99.2	97.1	97.8	98.9	**99.3**	**99.3**
	HI	92.8	91.8	90.9	92.3	94.5	94.5
	BR	92.8	95.4	95.1	79.7	95.7	**96.8**
	PR	88.6	85.5	88.9	89.6	**90.8**	90.5
	HeLa	97.3	92.2	95.2	94.8	97.7	**98.1**
	LE	99.0	97.3	98.1	97.6	98.8	**99.2**
	LT	98.7	97.1	98.1	98.0	99.0	**99.1**
	RNAi	**96.6**	94.1	92.8	90.4	95.7	**96.6**
	Average	95.4	92.8	93.5	90.4	95.5	**96.4**
***LPQ***	PAP	90.2	90.1	88.1	74.6	87.4	**90.9**
	VIR	94.9	93.7	91.7	87.0	94.8	**96.2**
	CHO	99.2	97.6	98.4	98.9	**99.4**	99.2
	HI	92.0	93.0	92.0	92.0	94.7	93.9
	BR	95.7	96.4	**96.9**	81.5	95.9	96.8
	PR	86.2	86.7	90.6	87.2	**91.7**	91.2
	HeLa	97.2	94.9	96.0	92.9	97.6	**97.9**
	LE	97.6	97.1	97.2	96.0	98.6	**98.7**
	LT	97.7	97.5	98.0	98.3	**98.9**	98.7
	RNAi	95.2	91.4	94.7	91.5	**95.8**	95.6
	Average	94.6	93.8	94.4	90.0	95.5	**95.9**

The results reported in Table 14 showed that the region-based approaches outperformed, with a *p*-value of 0.05, the standard application of texture descriptors. Especially, compared to the baseline *O*, *All+O* improved *AUC* (or did not perform worse) for all the datasets and for the three tested descriptors with a *p*-value = 0.05.

**Table 14:** *AUC* obtained using the region-based approaches and *LTP*, *RICLBP* and *LQP*.

		Region-based approach							
Descriptor	Dataset	*O*	*Edge*	*Saliency*	*Wavelet*	*DoG*	*Saliency+Wavelet*	*All*	*All+O*
***LTP***	PAP	91.4	87.8	89.3	87.7	86.6	88.9	89.0	**91.7**
	VIR	93.5	94.0	93.4	93.5	**94.9**	94.3	94.4	94.1
	CHO	99.9	99.9	**100**	99.9	99.9	99.9	99.9	99.9
	HI	91.6	92.7	90.6	92.4	92.6	92.7	**92.8**	92.3
	BR	96.9	96.4	96.1	96.2	95.9	96.5	96.6	**97.6**
	PR	89.7	87.0	90.3	87.8	85.1	89.7	90.0	**93.2**
	HeLa	98.6	98.6	98.6	98.5	98.6	98.8	**98.9**	98.7
	LE	99.5	99.5	**99.7**	99.6	99.3	**99.7**	**99.7**	99.6
	LT	99.3	99.3	**99.6**	99.4	99.4	**99.6**	**99.6**	99.5
	RNAi	97.0	97.0	**97.4**	97.0	96.6	97.3	97.2	97.3
	Average	95.7	95.2	95.5	95.2	94.9	95.7	95.8	**96.4**
***RICLBP***	PAP	91.8	92.0	92.5	91.9	93.3	92.7	**92.7**	92.4
	VIR	97.6	97.7	97.4	97.5	**97.8**	97.7	97.7	**97.8**
	CHO	99.2	99.8	**99.9**	99.7	99.8	99.8	99.8	99.7
	HI	92.8	93.7	93.4	93.6	93.8	93.9	**94.0**	93.5
	BR	92.8	93.8	94.0	94.6	93.9	95.0	**95.0**	93.9
	PR	88.6	89.3	89.2	88.5	88.0	**89.6**	**89.6**	89.4
	HeLa	97.3	98.4	**98.7**	98.2	98.8	98.6	98.6	98.2
	LE	99.0	99.5	**99.6**	99.3	**99.6**	99.5	99.5	99.4
	LT	98.7	99.1	**99.4**	98.5	99.3	99.2	99.2	99.0
	RNAi	96.6	96.8	**97.4**	96.8	97.2	97.2	97.1	96.9
	Average	95.4	96.0	96.2	95.9	96.2	**96.3**	96.3	96.0
***LPQ***	PAP	90.2	89.3	90.8	90.4	90.3	**90.9**	**90.9**	90.7
	VIR	94.9	94.4	94.5	94.7	94.1	**95.2**	**95.2**	**95.2**
	CHO	99.2	99.6	**99.8**	99.6	99.6	**99.8**	**99.8**	99.6
	HI	92.0	92.9	92.7	92.8	92.5	**93.2**	**93.2**	92.7
	BR	95.7	97.3	96.5	96.2	96.1	96.5	**96.8**	96.3
	PR	86.2	88.7	**90.5**	88.9	86.6	90.3	90.2	88.7
	HeLa	97.2	98.0	**98.5**	98.0	98.2	98.4	98.4	98.0
	LE	97.6	98.2	**99.4**	98.1	98.7	98.8	98.7	98.2
	LT	97.7	98.4	**99.2**	97.6	98.8	98.6	98.8	98.3
	RNAi	95.2	94.9	**95.7**	94.5	95.5	95.1	95.2	95.3
	Average	94.6	95.2	**95.8**	95.1	95.0	95.7	95.7	95.3

Finally, to summarize the best techniques presented in this section, we created a further ensemble *F* where we combine *Full_Bsif*, *O+Wa+OR(LTP)*, *O+Wa+OR(RICLBP)*, *O+Wa+OR(LPQ)*, *All+O(LTP)*, *All+O(RICLBP)* and *All+O(LPQ)*. *F* was compared in Table 15 to *Full_Bsif*: *F* performed better than *Full_Bsif*, or at least equally, on all the additional datasets with a *p*-value of 0.05.

**Table 15:** Comparison of *Full_Bsif* and the ensemble *F* of the best methods investigate in this section (*AUC* is reported).

	*Full_Bsif*	*F*
PAP	91.4	**93.6**
VIR	97.0	**98.1**
CHO	**99.9**	**99.9**
HI	94.0	**94.8**
BR	96.7	**97.5**
PR	91.9	94.5
HeLa	**99.2**	**99.2**
LE	99.8	**99.9**
LT	**99.8**	**99.8**
RNAi	**98.2**	**98.2**
Average	96.8	**97.6**

## Conclusions

In this work, we assembled and tested many state-of-art texture descriptors and a large set of preprocessing methods for demanding image classification tasks and their application in a new and very specific biological problem, i.e. the automatic assessment of the maturation level of hPSC-RPE cells. This is a very well warranted problem as RPE cells are planned to be used for implantation and for *in vitro* cell models for drug and disease modeling. In all these applications the perquisite for the maturation assessment is the non-invasiveness and consequently there is the need for label-free methods. Thus, analysis methods based on just phase contrast microscopy images are welcomed.

The first aim of this work consisted in applying three new methods to create ensembles of texture descriptors (based on combinations of preprocessing techniques, region-based approaches and *Bsif* with different filter sizes and binarization thresholds) to find the most suitable descriptors for the texture-based classification of the considered datasets, in particular of the RPE dataset. A combination of different preprocessing techniques (i.e. *Wa*, *OR* and *Ga* applied at three different scale of representation obtained by *MRS*) allowed to boost all the best performing descriptors (see Table 9, with the exception of *WLD*, for which *MRS* was not necessary). It is interesting to note that while each descriptor obtained the best performance with a different preprocessing, the fusions *Wa*+*OR*+*Ga* and *Comb*, improved the single best preprocessing for all the descriptors. Similar improvements were obtained by the region-based methods, in particular by combining the region selection by *Edge*, *Wavelet* and *Saliency* (see Table 10). However, it is interesting to note that a global combination of preprocessing and region-based feature extraction did not provide significant improvements compared to using the two approaches individually (see Table 10, last column). Therefore, we chose to exploit only preprocessing, hence obtaining the new ensemble *Comb(RICLBP)* + *Full_Bsif* + *MLPQens* + *MLCP*. This approach performance was *AUC* = 86.49%, which is the best result observed on the RPE dataset.

The second aim of this work was to provide the methodological core for a software tool in order to assess quantitatively the level of development of hPSC-RPE cells, compared to the classification provided in [4,62]. Our study primarily shows that a computer vision system is able to classify the RPE cell maturation stage and, secondly, it enables a correct and repeatable estimation of the maturation level on new images, a necessary step before using these specific cells and tissues, e.g. for drug testing. The most accurate ensemble, *Comb(RICLBP)* + *Full_Bsif* + *MLPQens* + *MLCP*, got an *AUC* of 86.49%, confirming that image processing methods can be employed to classify the maturity of RPE in microscopy images. Moreover, we proved that using the higher-performance, but slower to be validated, leave-one-image-out classification protocol we obtained for the best method *Comb(RICLBP)* + *Full_Bsif* + *MLPQens* + *MLCP* AUC over 91%.

The only related studies are [9] and [11]. Jiang *et al*. [9] reported a correlation between two specific morphological features, cell area and aspect ratio, two maturation stages (young, <61days-old *vs* old, ≥100 or 180 days-old) and two genotypes (control *vs* rd10). However, the cell source was different, mouse eyes *vs* RPE cells derived from hPSCs, as well as the maturation stages, since their focus was only in the cobblestone stage. In Kamao *et al*. [11], the degree of pigmentation (objective dPG, in the original publication) was assessed as the main marker on hPSC-RPE maturation, by means of a manual assessment of the RGB values from the Photoshop's Info Palette, for single cells (obtained by manual segmentation) and cell-groups. In spite of the findings, in particular that the objective dPG correlated with the RPE function, the technique of Kamao *et al*. [11] required user interaction, which might not be objective and is not suitable for huge number of images.

To prove the feasibility of the proposed methods not only on RPE images, but also for a wider range of biological applications, additional tests were performed on a selection of 10 datasets, spacing from diagnostic to microscopy images (electronic transmission as well as fluorescence imaging). We observed that the best-performing configurations of the three new proposed approaches (namely region-based descriptors, the ensemble of preprocessing algorithms and the improved *Bsif*) provided good classification results, obtaining an average *AUC* greater than 95%. In particular, we compared all the tested/proposed ensemble approaches and the best method resulted to be *Full_Bsif* that outperformed all the other approaches with a *p*-value of 0.05. This result highlights the key role of these methods in improving many texture descriptors’ accuracy in the classification of different kind of biological images. Of note, the effectiveness of *Full_Bsif* has to be seen all over the tested datasets (see Table 12). By changing the filter size and the binarization threshold we can obtain *Size_Bsif* and *Full_Bsif* which work better than the baseline *Bsif* on all the tested datasets (see Tables 8 and 12). On the RPE dataset, *Full_Bsif* by itself obtains results lower than RICLBP+MLPQens+MLCP (82.79% vs 84.60%), nevertheless *Full_Bsif* obtains statistically higher performances on the additional datasets, thus resulting the best ensemble based on a single descriptor. Finally, we tested on the additional datasets, a last ensemble *F* built by gathering the best techniques of the aforementioned three new approaches: *Full_Bsif*, *O+Wa+OR(LTP)*, *O+Wa+OR(RICLBP)*, *O+Wa+OR(LPQ)*, *All+O(LTP)*, *All+O(RICLBP)* and *All+O(LPQ)*. *F* outperformed *Full_Bsif* in six datasets and obtained equal AUC in the other four. Due to the variety of the additional datasets, *F* represents our proposed ensemble to process a generic dataset as well as a suggestion to other researchers for further studies on this topic.

Of note, the approaches investigated in section 3.2 showed better performances on the additional datasets than in the RPE dataset. This is due to the nature of each dataset, e.g. staining, microscopy technique, etc. The RPE dataset was acquired directly imaging the cell cultures through a phase-contrast microscope. On the other hand, among the additional datasets we had more complex imaging techniques such as electronic transmission (VIR) or fluorescence imaging (CHO, HeLa, LE, LT, RNai). Such techniques involve sample treatment (e.g. ultra-thin samples for electron transmission, staining with antibodies for immunofluorescence or other stains for histology images). In spite of the better image quality which then affects the classification performance, such processing necessarily alters the samples and it is time-consuming. Moreover, many datasets provided pre-segmented images, thus excluding the textural information from the background.

The main limitation in the RPE study is the strict standards we had to define and fulfill for the image acquisition, necessary to build a reliable dataset, but demanding to be implemented in the laboratory practice.

As future work, we will build new methods to build region-based approaches for the classification of biological image datasets. Furthermore, we aim to use a heterogeneous ensemble of classifiers, instead of a stand-alone *SVM*, to improve the performance. Finally, to remove potentially confounding patterns, we plan to design an automatic method to remove the background from samples such as fusiform and epithelioid images.

In conclusion, in this paper we presented three methods to developed ensembles of texture descriptors, proving that specific preprocessing and ensembling techniques improve the performance of many state-of-art texture descriptors. Moreover, we automated the classification of the maturation stages of RPE cells by means of an ensemble of texture descriptors. Such methods were finally validated on a wide set of general biological image analysis problems.

## Abbreviations

All: fusion by sum rule among Saliency, Edge and Wavelet
AUC: area under the ROC curve
BR: mammography dataset
Bsif: binarized statistical image features
CHO: chinese hamster ovary cell dataset
CLBP: completed LBP
CoALBP: multi-scale co-occurrence of adjacent LBP
COLORS: Set of statistical descriptors computed on the RPE color images.
Comb: fusion by sum rule among Wa, OR and Ga. Preprocessing applied to the original image and the two MRS images.
DENSE: multi-scale densely sampled complete LBP
DENSEri: multi-scale densely sampled complete rotation invariant LBP
DoG: difference of gaussians
dPG: degree of pigmentation
Edge: Edge variant of a texture descriptor
F: ensemble of the best methods tested on the additional datasets (see section 3.2)
Full_Bsif: ensemble based on Bsif with variable filter sizes and thresholds
GA: Gabor filter applied to the original image
HeLa: 2D HeLa dataset
hESC: human embryonic stem cells
HI: hystopathology dataset
hiPSC: human induced pluripotent stem cells
HOG: histogram of oriented gradients
hPSC: human pluripotent stem cells
LBP: local binary pattern
LBP-HF: multi-scale LBP histogram Fourier features
LBPri: rotation invariant LBP
LCP: local configuration pattern
LCPri: rotation invariant LCP
LE: locate endogenous mouse sub-cellular organelles dataset
LQP: local quinary pattern
LT: locate transfected mouse sub-cellular organelles dataset
LTP: local ternary pattern
LTPri: rotation invariant LTP
MLCP: multi-quinary coding version of LCP
MLPQ: multi-ternary coding version of LPQ
MLPQens: ensemble of MLPQ
MLQP: multi-threshold version of LQP
Morph: Strandmark morphological features
MRS: gaussian scale space representation
NTLBP: multi-scale noise tolerant LBP
O: features extracted only from the original image
OR: features extracted from the three orientation images obtained from the original image
PAP: Pap test dataset
PR: protein dataset
RICLBP: multi-scale rotation invariant co-occurrence of adjacent LBP
RNAi: RNA dataset
RPE: retinal pigmented epithelium
Saliency: saliency map
SFFS: sequential forward floating selection
SFS: sequential forward selection
Size_Bsif: ensemble based on Bsif with variable filter sizes
SVM: support vector machine
TEO: Finnish National Authority for Medicolegal Affairs
Wa: features extracted from the four images obtained by wavelet decomposition
WaH: features extracted from the images obtained a two level wavelet decomposition
Wavelet: wavelet decomposition into horizontal, vertical and diagonal coefficients
WLD: Weber law descriptor
VIR: virus dataset
